# Vaccine equity implementation: exploring factors influencing COVID-19 vaccine delivery in the Philippines from an equity lens

**DOI:** 10.1186/s12889-024-20578-7

**Published:** 2024-11-05

**Authors:** Junqiang Zhao, Shishi Wu, Renz Andrew Rafal, Helena Manguerra, Quanfang Dong, Hongyu Huang, Lincoln Lau, Xiaolin Wei

**Affiliations:** 1 Waypoint Research Institute, Waypoint Centre for Mental Health Care, Penetanguishene, ON Canada; 2 Dalla Lana School of Public Health, University of Toronto, Toronto, ON Canada; 3 International Care Ministries, Manila, Philippines

**Keywords:** Vaccine equity, Health equity, Vaccine ethics, Vaccine hesitancy

## Abstract

**Background:**

During the early phase of the COVID-19 vaccine rollout, low and middle-income countries (LMICs) were facing challenges in achieving equitable vaccine delivery. Few studies have contextualized global vaccine distributive injustice into national-specific contexts to understand its impact on vaccine delivery from an equity perspective. We aimed to investigate factors influencing equitable COVID-19 vaccine delivery in the Philippines and to provide recommendations to enhance equitable vaccine delivery in LMICs to prepare for future health emergencies.

**Methods:**

The Health Equity Implementation Framework was employed to guide this qualitative study. We recruited participants using purposeful and snowballing sampling strategies. Semi-structured interviews were conducted with participants in person, online, or over the phone. A reflective thematic analysis approach was employed to analyze data.

**Results:**

We recruited 38 participants including seven high-level stakeholders from the public and private sectors, 14 health workers, and 17 community members in the province of Negros Occidental, Philippines. Equitable delivery of COVID-19 vaccines was influenced by an interplay of multiple factors operating in different domains. Contextually, the rapidly evolving nature of the COVID-19 virus, ongoing scientific advancements, and international negotiations directed national-level vaccine policies. Political commitment and support were recognized as crucial drivers for successful vaccine delivery, with a strong emphasis on health information framing and communication and adherence to human rights principles. The vulnerability of the health system significantly impacted the timely and effective distribution of vaccines. Furthermore, the geographical characteristics of the Philippines presented unique logistical challenges to vaccine delivery. At the recipient domain, individual perceptions of vaccines, shaped by their socioeconomic status, exposure to (mis)information, social influence, and entrenched religious beliefs, played a major role in their vaccine decisions and thus vaccine coverage regionally. Additionally, vaccine characteristics and operational challenges related to its distribution also impacted fair allocation.

**Conclusions:**

The findings highlight the urgent need for LMICs to strengthen their health system resilience and sustainability and use multilevel strategies to build public trust to improve vaccine uptake and coverage. Moreover, each LMIC must be attentive to its unique contextual factors to develop tailored implementation strategies to promote equitable vaccine distribution.

**Supplementary Information:**

The online version contains supplementary material available at 10.1186/s12889-024-20578-7.

**Table:** 

**Text box 1. Contributions to the literature**
• Existing literature on vaccine equity predominantly focuses on global-level distributive justice with limited research exploring the country-specific context that affects local vaccine equity, particularly in LMICs. Our study contributes to this field by examining factors influencing equitable COVID-19 vaccine delivery in the Philippines.
• The qualitative study drawing on the Health Equity Implementation Framework contributes to implementation science by identifying factors for vaccine equity implementation and potential implementation strategies.
• The findings revealed common barriers to vaccine equity in LMICs, such as health system vulnerability and vaccine hesitancy due to socioeconomic status, and barriers specific to the Philippines, such as geographical disparities and religious beliefs among ethnolinguistic groups.

## Background

### Vaccine equity in low- and middle-income countries (LMICs)

Vaccination has been proven to be one of the key strategies to prevent death and severe consequences from COVID-19 infection [1–4]. Over the past two years, extensive efforts have been undertaken at the global, national, and local levels to facilitate the rollout of COVID-19 vaccines. As of June 2023, over 13 billion doses have been administered globally [5]. Despite the demonstrated effectiveness and the remarkable achievements in vaccine rollout, there had been significant challenges in distributing vaccines equitably around the world, particularly during the early stage of the pandemic [6, 7]. High-income countries have had better access to vaccines and have been able to vaccinate a larger proportion of their populations at an early stage, while LMICs have faced significant challenges in obtaining sufficient supplies [8] and suffered a disproportionately higher burden of the pandemic compared to their high-income counterparts [9]. The phenomenon of vaccine nationalism has not only resulted in significant surges in infections within countries with low vaccine availability [10], but also adversely affected the global public health crisis management [11]. To address the issue of vaccine equity, the World Health Organization along with other partners, launched the COVAX initiative aiming to provide equitable access to COVID-19 vaccines for LMICs with the guiding principle “no one is safe, until everyone is safe” [12]. However, regional disparities in vaccine distribution persist.

### Vaccine equity in the Philippines

In the Philippines, vaccine rollout has been ongoing since March 2021 [13]. As of March 2023, over 189.32 million doses have been administered, with 78.44 million people fully vaccinated [14]. Despite the increasing vaccination rates and the enormous efforts from the government to promote equitable vaccine delivery [15], achieving vaccine equity (i.e., everyone, regardless of their socioeconomic status, geographic location, or background, has fair and just access to vaccines [16]) was still challenging, particularly during the early stage of the vaccine rollout when the country had a limited supply. The issue of vaccine equity requires careful attention and deliberate actions to provide valuable insights for future public health emergencies and vaccination campaigns.

In the Philippines, COVID-19 has disproportionately impacted the socioeconomically disadvantaged communities including seniors, individuals living in poverty, indigenous groups, and people with disabilities [17–19]. According to the latest data from the Department of Health in the Philippines, as of June 2023, people aged 50 and above made up a significant majority (71.2%) of the total mortalities associated with COVID-19 [20]. A policy analysis study indicated that while several policy documents mentioned financial support and accommodations for individuals with disabilities during the pandemic, only a few provided specific details on program implementation and monitoring [21]. A survey conducted by the World Bank among low-income households in the Philippines demonstrated a significant decline in visits to health centers for children aged five and below during the lockdown, dropping from 73% before the lockdown to 41% during the lockdown [22]. The Philippines has around 14 – 17 million indigenous peoples, part of approximately 110 ethnolinguistic groups, who heavily depend on farming, fishing, and crafting as their primary means of livelihood. The implementation of lockdown measures and restrictions on people’s mobility have significantly hindered their access to water, energy, food, and healthcare [19].

Given these concerns, it has become imperative to investigate factors impacting COVID-19 vaccine delivery from an equity standpoint in LMICs like the Philippines. However, existing literature on vaccine equity predominantly focuses on global-level distributive justice [6–8, 23, 24] with limited research exploring the country-specific context that affects local vaccine equity, particularly in LMICs. A recent systematic review examined the impact of geographic, demographic, and sociodemographic characteristics on vaccine equity in LMICs [25]. Nevertheless, it failed to capture the contextual and process-oriented factors that significantly impact vaccine delivery. In the Philippine context, most studies on COVID-19 vaccines have primarily centered on vaccine attitudes and factors related to hesitancy and confidence [26–30] with no studies that investigated factors impacting its equitable delivery. To our knowledge, very limited studies have specifically investigated factors influencing equitable vaccine delivery during the pandemic in LMICs.

This study, therefore, aimed to examine the factors influencing COVID-19 vaccine delivery in the Philippines from an equity lens by exploring stakeholder perspectives and provide recommendations to enhance equitable vaccine delivery in LMICs in response to future public health emergencies. These efforts are crucial to shed light on the challenges and opportunities associated with implementing equitable vaccination programs and inform the development and customization of implementation strategies to promote equitable vaccine distribution.

## Methods

This was an interpretive descriptive study [31, 32] informed by the Health Equity Implementation Framework [33]. Interpretive descriptive design is appropriate for this study as our research aimed to understand the influencing factors of COVID-19 vaccine delivery in the Philippines, which *“contributes directly to our understanding of how people experience their health and illness (P.173)”* [31]. The Health Equity Implementation Framework is a useful framework to guide this study in that it delineates factors across three domains — context, recipients, and innovation characteristics — that are crucial for predicting equitable implementation and highlights the importance of addressing social determinants of health and culturally relevant factors of recipients for achieving health equity [33, 34]. By employing this framework to guide our data collection and analysis, we aimed to achieve a thorough and nuanced understanding of the factors that influence equitable vaccine delivery. This study received ethical approval from the University of Toronto Research Ethics Board (# 00042287) and the International Care Ministries ethics committee in the Philippines (# 0001). We referred to the Standards for Reporting Qualitative Research to guide the reporting of findings [35].

### Setting

This study was conducted in the province of Negros Occidental, Philippines. Negros Occidental, located in the Western Visayas of the Philippines, is the most populous province in the region, with nearly 3.2 million residents according to the 2020 census [36]. The majority of the population here adheres to Christianity, with Roman Catholicism being the predominant religion. Specifically, we recruited and interviewed participants from Bacolod City and Bago City. Bacolod City, the capital of Negros Occidental, is the most urbanized area in the province, with a population of about 600,000, accounting for about a quarter of the province’s total population [37]. However, in 2021, the poverty incidence in Bacolod was as high as 19% despite the city’s resources and economic opportunities [38]. Bago City, located about 20 km from Bacolod City, has a population of 191,210 as of 2020. The city remains predominantly agrarian, with substantial income derived from the production of sugar and rice [39].

### Sampling strategy

The inclusion criteria for participants were stakeholders who were involved in designing, planning, or implementing COVID-19 vaccination programs in the Philippines, including decision-makers of vaccination programs, program administrators, health workers, staff from Non-Governmental Organizations (NGOs), private organizations, and community leaders.

We used a two-stage method to sample key informants for interviews. Purposive sampling was used to identify appropriate key informants from existing networks and contacts of the principal investigator, local partners, and co-investigators. We obtained a list of potential key informants through the discussion among the investigators and our collaborators in the Philippines. Prospective interviewees were contacted via email, Zoom, or phone by local field workers who explained the research project and the purpose of the research, and sought initial consent for the interview. A formal letter of invitation was sent to prospective interviewees who provided their initial consent for the interview via email. We then used a snowball sampling strategy to identify other potential candidates until we gained a sense of data sufficiency.

### Data collection

Interviews were conducted in July 2022, mostly in-person, but also online or over the phone depending on the availabilities and preferences of interviewees. Interviews were conducted in the preferred language of the interviewee. The interviews began with an explanation of the study, followed by the delivery of a verbal consent script and signing of the informed consent document for both study participation and audio recording. For those participants who consented to participate in the study without any audio recording, we sought consent to take notes during the conversation.

We developed three interview guides based on the Health Equity Implementation Framework, for high-level stakeholders who were involved in the decision-making of COVID-19 vaccine programs, implementers of COVID-19 vaccine programs (mainly health workers), and vaccine recipients (mainly community members) (see appendix). All interview guides included questions inquiring about challenges and facilitators of COVID-19 vaccine equitable delivery and strategies to improve vaccine acceptance and uptake among vulnerable populations. The three interview guides have minor differences with one focusing on political and policy level considerations, one on vaccine rollout logistics, and the other on recipients’ perspectives. The interview guides were pilot-tested for clarity before being used with our participants.

### Data processing and analysis

Audio recordings were transcribed verbatim (and translated if needed) by two local bilingual staff from the Philippines. A reflective thematic analysis approach [40] was used to analyze the data by team members (JZ, RR, &SW) independently and collaboratively. We imported the transcripts to NVIVO version 12 software [41] and analyzed the data with the following five steps. (1) Familiarization. We read the transcripts thoroughly to immerse ourselves into the text and noted down initial ideas on the factors that influenced vaccine equity in the Philippines; (2) Generating codes. Each data analyst used an inductive approach to systematically identify and label meaningful units of data that are relevant to vaccine equity. (3) Constructing themes. Three data analysts met to group codes, identify recurring patterns, and build candidate themes. (4) Refining and defining themes. We compiled all coded data for each of the candidate themes and reviewed them to ensure that the data related to a central organizing concept, and further refined the themes as necessary. We defined all these themes in the context of the equitable COVID-19 vaccine delivery; (5) Building a visual model. We mapped the themes into the three dimensions of the Health Equity Implementation Framework and constructed a visual model to illustrate their relationships.

## Results

A total of 38 participants were interviewed, including seven high-level stakeholders from the public and private sectors, 14 health workers, and 17 community members (Table 1).

**Table 1:** Demographic characteristics of participants

Variables	*N* (%)
Total number	38 (100.0%)
City health office health workers	8 (21.1%)
Barangay health workers & volunteer workers	6 (15.8%)
High-level stakeholders in the public & private sectors	7 (18.4%)
Community members	17 (44.7%)
Sex	38 (100.0%)
Male	9 (23.7%)
Female	29 (76.3%)
Age^a^	30 (100.0%)
20 ∼ 29	3 (10.0%)
30 ∼ 39	8 (26.7%)
40 ∼ 49	2 (6.7%)
50 ∼ 59	12 (31.6%)
60 ∼ 69	5 (16.7%)
Education^a^	29 (100.0%)
Primary school	3 (10.3%)
Secondary school	1 (3.4%)
High school	3 (10.3%)
College	17 (58.6%)
Master	1 (3.4%)
PhD	4 (13.8%)

^a^ variables contain missing values

### Factors influencing COVID-19 vaccine delivery

**Fig. 1:**
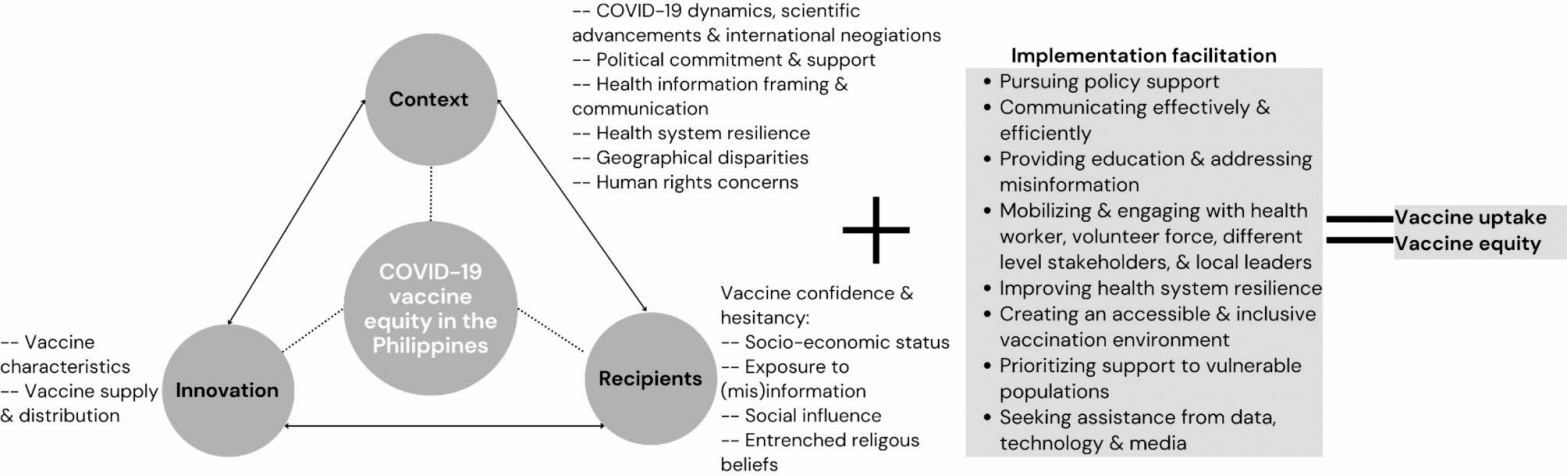
COVID-19 Vaccine Equity Implementation in the Philippines

Guided by the Health Equity Implementation Framework, we found that the delivery of COVID-19 vaccines in the Philippines was influenced by a complex interplay of factors operating in three domains (Fig. 1). Specific descriptions, definitions, and corresponding quotes for factors at context, innovation, and recipients’ domains are provided in Table 2.

**Table 2:** Factors influencing COVID-19 vaccine delivery

Factors	Definition in context	Quotes
**Context-related factors**		
COVID-19 dynamics, scientific advancements and international negotiations	The evolving nature of the COVID-19 virus, including its transmission patterns and mutation variants, the scientific progress made in understanding and combating the virus, and the international negotiations on vaccine allocation directed vaccine policies and thus its delivery in the Philippines.	*“So*, *we have this pattern*, *we would shut it down for about a month to keep it down. But when we released the lockdown*, *what would happen is that Filipinos would return home to their provincial homes*, *and then they would carry the variant with them*, *which is why we decided to prioritize the National Capital Region and the surrounding provinces in order to build an immunity wall to prevent variants from entering”* *P21 (High-level stakeholders in the private sector).* *I think that was October. During that time*, *that was a challenge because COVID-19 was increasing. We (the province) created the emergency operation chapter. That would be composing the governor and of course the responsibility is at the provincial health officer as the vice chair and the action officer of the operation chapter.* *P18 (Provincial health officer)*
Political commitment and support	Political leaders’ commitment and their policies on vaccine distribution, including vaccination priority policies, funding allocations, and decision-making processes directly influenced the allocation of resources and the efficiency of vaccine delivery.	*“Our local government executive was really supportive on us and every time there were requests for the vaccines …he would communicate with the governor and the provincial administrator if they could allocate more vaccines to our city. He has really extended his limits… exhausting all things possible to be able to cater to our requests”* *P3 (Volunteer nurse-vaccination data manager)* “*The least that you can do is limit access to the basic needs of people so that they are forced to be vaccinated… That is not telling you that I will be jailing you*, *but you have to follow whatever policy*, *or whatever memo*, *or whatever executive order that will be issued. You cannot get permits in LGUs (Local Government Units) particularly cities and municipalities if you are not vaccinated. You cannot get your permit for driving tricycles. You cannot get permits for your business establishments… Likewise*, *you can never enter any establishment even if it’s a coffee shop*, *[or] a grocery if there is a law. In the LGUs*, *there is a policy that exists on the limitation for those unvaccinated individuals. That is one way of convincing them.”* *P18 (Provincial health officer)*
Health information framing and communication	Effective presentation, dissemination, and communication of vaccine information to the public shaped public perceptions of vaccine safety and effectiveness. Moreover, timely and efficient information exchange among vaccination teams fostered mutual understanding and coordination, which enhanced vaccination efforts.	*“In terms of getting information for the COVID-19*, *The Department of Health Facebook page is really helpful … just like talking to the neighbors”* *P3 (Volunteer nurse-vaccination data manager)* *“There are those who threaten people saying that this is the last vaccine they have*, *no more vaccine. If you want to have the vaccine later*, *you [must] pay for yourself. That’s why so many people went to the mall.”* *P35 (Community member)* *“Almost monthly*, *the central office is conducting meetings and orientations with the different stakeholders in the regional offices*, *in the provincial health offices to include the stakeholders and our cold chain hubs in order for us to be informed on the new updates. The central office has also provided a google drive where you can also see the Google sheet and be updated on the data and policies that are being implemented right now”* *P10 (Public health pharmacist)*
Health system resilience	The capacity of health systems including the availability and adequacy of healthcare professionals and resources, governance, coordination, preparedness and planning was essential for an effective response to vaccine distribution.	*“Actually*, *there is a shortage of health workers. And not just health workers*, *there is a shortage in the mobilization*, *in the vehicle that they are going to use. First*, *a lot of health workers in the provinces are busy for other deliverables. They are facilitating the vaccination rollout. So it is really important if we have one specific person that is handling*, *overseeing*, *and monitoring the covid-19 vaccines. However*, *due to a shortage of health personnel and staff*, *it is one of our challenges here in our region. Next is mobilization. One of the reasons why there is a late delivery or the unavailability of vaccines in the area is due to the unavailability of the vehicle”.* *P10 (Public health pharmacist)*
Geographical disparities	The unequal distribution of healthcare resources and vaccines due to geographic locations impacted vaccine delivery and uptake regionally.	“*You cannot force the farmers and those living in mountainous areas*, *because they say they don’t go down the city.”* *P7 (Public nutrition scholars)* *“Because here in the Philippines*, *some are very poor. Even you are in this barangay*, *some other people are still living in the mountains. And some of them could not afford to pay for their transportation. So*, *that’s the time that the vaccinators go house to house.”* *P4 (Volunteer nurse)*
Human rights concerns	The ethical and legal considerations regarding human rights and freedom impacted vaccine policies and vaccine distribution.	*“You know we have human rights*, *you cannot just implement any provision*, *any law that violates human rights. When we say*, *we have the right to refuse vaccination*, *we have that right. They can always file cases against you*, *most especially if lawyers are anti-vax.”* *P18 (Provincial health officer)*
**Vaccine-related factors**		
Vaccine characteristics	The attributes and qualities of COVID-19 vaccines, including efficacy, safety, storage requirements, dosage schedules, and eligibility criteria impacted vaccine delivery, administration and uptake.	*“Initially when the pandemic starts*, *there are many brands of vaccines that are being delivered by the central office. One of the challenges is that vaccines near expiry are delivered to our area. Due to the slow consumption of the COVID-19 vaccines in the region*, *it is one of the causes why there are lots of vaccines that are expired”.* *P10 (Public health pharmacist)*
Vaccine supply and distribution	The practical challenges including transportation, cold chain management, staffing, and other operational considerations impacted vaccine distribution and administration.	*“The main concern is the storage of the vaccine. Like what happened today*, *there was a delivery from the central office*, *from the DOH*, *and we have no more freezers to store them so*, *we have to start thawing some of the vaccines and prioritize these thawed vaccines for distributions”* *P15 (Cold chain storage in-charge regional health officer)*
**Recipient-related factors**		
Socioeconomic status	The economic and social factors, such as income, education, occupation, and access to healthcare influenced the uptake of vaccines within communities.	*“You cannot force the farmers and those living in mountainous areas*, *because they say they don’t go down to the city.”* *P8 (Public nutrition scholar)* *My mother who died at 96 in last September*, *was positive of COVID-19…The reason why she was not vaccinated was that it was hard for them to bring her to the mall because she can’t walk properly. And that time there was no mobile clinic.* *P24 (Community member)*
Exposure to (mis)information	The availability, accuracy, and comprehension of information related to COVID-19 vaccines, including those from mass media, social media, and other forms of communication had a substantial impact on individuals’ perceptions, attitudes, and behaviors regarding the vaccines.	*“--Where do the people get information about the vaccine*, *about COVID vaccines? --On Facebook…Everybody uses Facebook.”* *P19 (Health worker-vaccine admission and screening)* *“--Do you think people got a lot of information about the vaccines from online sources? --I think yes*, *especially in social media…There’s an instance in Manila where the child has cerebral [epilepsy] (after vaccination). And many people visit the site*, *other mothers are hesitant to bring their children to the vaccination site because of what they see on social media. They are discouraged.”* *P9 (City health office-health educator)*
Social influence	The social norms, values, and beliefs shaped vaccine distribution and acceptance among different communities and populations. It included the influence from community leaders, pastors, opinion leaders and champions, family and relatives, and other social networks.	*“Most who complain are senior citizens because as what they know*, *vaccines are poison to our body. They have negative thinking and mindset towards vaccines because of the information given by our doctors and nurses*, *they were convinced afterwards.”* *P13 (Public nutrition scholar)* *“Because most of them are listening to people talking about some adverse effects. Yeah. That’s Filipino culture. Gossip. Here in barangays*, *if one person in the household got sick after vaccination*, *everyone in the community would know that and be discouraged*.” *P5 (Volunteer nurse-vaccination process supervisor)* *Some pastors support (vaccination)*, *but there’re some other pastors coming from different churches*, *they don’t agree on vaccination. They said God is in control and they just refuse to acknowledge the responsibility. Yes*, *we can’t deny God’s sovereignty but still*, *we have the responsibility*. *P25 (Community member)*
Entrenched religious beliefs	The deeply ingrained religious beliefs shaped individuals’ perceptions, attitudes, and decisions regarding COVID-19 vaccines.	*Those who are religious leaders in our country*, *they didn’t want to get vaccinated. Even they will die*, *they will not. Even in the hospital (when) they are advised for blood transmission*, *they prefer to die than (get a blood transfusion) … They believe in God but in different ways.* P14 *(Department of Health representative --vaccination process supervisor)* *“No*, *I’m not afraid. It’s just my own not to have the vaccine because I only trust God since I am already old and I am all right and I will just accept whatever may happen.”* *P34 (Community member)* *“It’s really the mindset of the people in the community because I ask them maybe the transportation is a challenge to them. They said*, *it’s not a challenge because the government provides*, *the barangay provides them transportation that brings them to vaccination sites. For them*, *it’s really the mindset.”* *P13 (Public nutrition scholar)*

### Context-related factors

The rapidly evolving nature of the COVID-19 virus, ongoing scientific advancements, and international negotiations had directed the national vaccine guideline development that guided the vaccine allocation and distribution, including the prioritization groups and operational procedures. Given the “*urgency to give protection and additional protection…in this emergency situation*” (P15: Cold chain storage in-charge regional health officer), the COVID-19 vaccination policy was developed differently from other routine vaccination campaigns. Vaccination guidelines had undergone rounds of updates in response to the new variants and latest research findings. “*For the booster dose*, *before it was stated that 6 months after the last dose*, *then it was revised that it should be three months after the first and second of our shots. There are so many changes actually in the previous. It was amended. It was revised…”* (P14: Department of Health representative responsible for vaccination process supervision).

Political commitment and support were emphasized as key drivers for vaccine rollout. Participants highlighted how it influenced various aspects of vaccination delivery and operation, notably the vaccination prioritization framework which gave vaccination priorities to health workers (A1 group), senior citizens (A2 group), and persons with comorbidities (A3 group), the vaccine passport policy that required the proof of vaccination document for entry into non-essential venues, and various incentive strategies and transportation support for vaccination. Additionally, participants described the leadership roles played by local government executives in promoting the vaccination campaign and their efforts to secure more vaccines for their respective areas. “*Our local government executive was really supportive on us and every time there were requests for the vaccines …he would communicate with the governor and the provincial administrator if they could allocate more vaccines to our city. He has really extended his limits… exhausting all things possible to be able to cater to our requests*” (P3: Volunteer nurse-vaccination data manager).

Along with the political commitment and support was health information framing and communication that had influenced vaccine rollout. Participants commended the Department of Health’s Facebook page as “*very helpful…just like talking to the neighbors”* (P3: Volunteer nurse-vaccination data manager) which had improved public perception, understanding, and acceptance of vaccines. Meanwhile, they criticized the spreading of fake information. “*There are those who threaten people saying that this is the last vaccine they have*, *no more vaccine. If you want to have the vaccine later*, *you [must] pay for yourself”* (P35: Community member). In addition, this theme also included effective information exchange among vaccination teams to ensure mutual understanding and coordination. An exemplary case in another province was described where the central office had routine meetings and orientations with the stakeholders and used Google Sheets to share updates (P10: Public health pharmacist). Furthermore, participants expressed concerns about individual autonomy and freedom in relation to vaccine policies, particularly the implementation of vaccine passport policies. They stressed that “*we have human rights*, *you cannot just implement any provision*, *any law that violates humans’ rights*” (P18: Provincial health officer), acknowledging the need for vaccination campaigns to uphold human rights principles while promoting vaccine uptake.

The health system vulnerability emerged as another key factor that significantly impacted vaccine delivery in our study. Participants constantly highlighted the insufficiency of health workers, mobilization vehicles, and vaccine storage refrigerators that hindered vaccine rollout. “*In every LGU (Local Government Unit)*, *you are lucky if you have and can afford 10 nurses. Most often than that*, *LGU only has a maximum of 5. That’s what LGUs can afford to spend. I think that’s the barrier that we need to address. We are still facing that barrier up to now*” (P18: Provincial health officer). The decentralized health system in the Philippines seemed to further complicate the situation, with different capacities and resources available across local government units. Disparities were observed in the interviews, with some regions struggling with limited resources and inadequate facilities, while others were relatively better equipped. This related to another important factor influencing vaccine delivery — the geographical characteristics of the study setting. The geographical layout within Negros Occidental posed huge logistical challenges in delivering vaccines, particularly in some remote or hard-to-reach areas. This challenge is pertinent to the entire country of the Philippines, given its archipelagic nature. Participants mentioned the “*transportation support”*, *“mobile vaccination”* and *“house-to-house”* strategies to improve vaccine coverage among people in those remote areas.

### Vaccine-related factors

Participants raised public concerns about the effectiveness, safety, and potential side effects of vaccines which had an impact on individuals’ vaccine brand preferences and willingness to receive the vaccine. “*Sinovac is mild*, *while the Astra is strong. When I got this booster…I told them I want Astra to be my booster. I want to feel the same effects as my first and second dose*.” (P24: Community member). The supply and distribution of vaccines encountered a few logistical challenges. Participants underscored the issue of quick expiration dates for some vaccines, which created pressure for efficient administration within a limited timeframe. “*The number one challenge was (that) there were times the vaccines that were being delivered were already near to expire*” (P10: Public health pharmacist). The storage requirements, such as ultra-cold temperatures for certain vaccines, posed challenges for resource-constrained areas with limited refrigeration capabilities.

### Recipient-related factors

At the recipient domain, individuals’ vaccine confidence, shaped by their socioeconomic status, exposure to (mis)information, social influence, and entrenched religious beliefs, played a major role in their decisions to receive vaccines. Individuals with lower socioeconomic status faced barriers to accessing vaccines equitably. “Y*ou cannot force the farmers and those living in mountainous areas*, *because they say they don’t go down the city*” (P8: Public nutrition scholar). Low levels of health literacy and limited awareness about the importance of vaccination among socioeconomically disadvantaged individuals also hindered their engagement with the vaccination process. “*There’s a small number of them (indigenous population) … There’s a difficulty in understanding them because they speak a different language…it is hard for them to understand what vaccine is and how it works*” (P9: Health educator).

Exposure to the vaccine misinformation undermined participants’ confidence in COVID-19 vaccines. A large proportion of participants reported that they accessed COVID-19 vaccine information from social media, particularly Facebook. They described instances of adverse events and the previous dengue vaccine controversy, which had gone viral on Facebook and discouraged people from receiving COVID-19 vaccines. Furthermore, senior citizens, indigenous populations, and people residing in remote and mountainous areas had limited access to health information, making them more susceptible to misinformation and vaccine hesitancy. “(*For) the senior citizens of the mountainous area*, *they can’t afford to have a television*, *they only have a radio*” (P6: Volunteer nurse).

Social influence from family, peers, community, and cultural and religious groups, played a significant role in shaping individuals’ vaccine decisions. “(*In) Filipino culture*, *children usually are taking care of their mother*, *father*, *grandpa*, *and grandma. Usually*, *they are the ones who decide with regards to the vaccination status of the senior*” (P18: Provincial health officer). Furthermore, participants highlighted the impact of entrenched religious beliefs on their attitudes and behaviors towards vaccination. “*I just trust God. I don’t care whether I will die because I have nothing to do with that*” (P34: Community member).

### Implementation facilitation

In addition to the influencing factors, we also identified implementation strategies employed by the government to strengthen vaccine uptake. For example, during the initial vaccine rollout in 2020 when the COVID-19 vaccine supply was limited, the Department of Health prioritized vulnerable populations, including healthcare workers, the elderly, and individuals with comorbidities for vaccination. To reach families in rural areas, barangay health workers were enlisted to conduct home visits and provide health education. Moreover, temporary vaccine sites were established in malls and stadiums to make it more convenient for the public to get vaccinated. These strategies bolstered vaccine acceptance and equitable distribution, thereby ensuring a more inclusive and effective vaccination program in the Philippines. Additionally, study participants also proposed strategies likely to facilitate equitable vaccine delivery based on their experiences of delivering COVID-19 vaccines. Participants emphasized the importance of addressing misinformation in communities and on social media. They highlighted the potential of engaging trusted community-based partners, such as community volunteers, health educators, or faith leaders, as information channels to disseminate reliable information about COVID-19 vaccines.

## Discussion

Based on the interview with 38 vaccine delivery stakeholders, we revealed that equitable vaccine delivery in the Philippines was influenced by a range of interconnected factors operating in different domains. In the contextual domain, the rapidly evolving nature of the COVID-19 virus, ongoing scientific advancements, and international negotiations directed national vaccine policies, which in turn, influenced local vaccine distribution strategies. Political commitment and support were recognized as crucial drivers for successful vaccine delivery, with a strong emphasis on health information framing and communication and adherence to human rights principles. The resilience of the health system significantly impacted the timely and effective distribution of vaccines. Furthermore, the geographical characteristics of the Philippines presented unique logistical challenges to vaccine delivery. In the recipient domain, individual perceptions of vaccines, shaped by their socioeconomic status, exposure to (mis)information, social influence, and entrenched religious beliefs, played a major role in their vaccine decisions and thus vaccine coverage regionally. Additionally, vaccine characteristics and operational challenges related to its distribution also impacted fair allocation.

Our study highlighted that the vulnerability of the health system in the Philippines posed a major obstacle to vaccine delivery. Despite the significant achievement in universal health coverage in 2019 in the Philippines, persistent challenges such as fragmented care, uneven distribution of health facilities, limited health human resources, and inadequate financing continue to impact the overall functioning and effectiveness of its healthcare system [42–44]. According to the Philippines Health System Review issued by the World Health Organization, the distribution of healthcare resources varies significantly across the country with almost two-thirds of hospital beds being in the island of Luzon, which includes the National Capital Region [45]. The same goes for the doctor-to-population ratio. The National Capital Region had a higher ratio of 10.6 doctors per 10,000 people, while regions such as Western Visayas (Region VI) and the Autonomous Region in Muslim Mindanao had lower ratios of 3.1 and 0.9 doctors per 10,000 people, respectively [45]. Furthermore, a 2020 report by the Philippine Institute for Development Studies revealed that approximately 75% of cities and municipalities across the country had an insufficient number of healthcare workers [46]. All these challenges have undermined the capacity of the Philippines’ health system to respond to the COVID-19 pandemic effectively and roll out COVID-19 vaccines nationwide promptly [47, 48]. Studies in other countries also corroborate this point. A recent systematic review on inequality in the distribution of the COVID-19 vaccine also found that national infrastructure and health system, such as the appropriate information system, functional cold chains in vaccine transport, medical and non-medical facilities per capita, healthcare access and quality, contributed to vaccine inequity [24]. Perception of the healthcare system as being inefficient and inflexible has also been identified as a hindrance to vaccine rollout in the Philippines [26].

Our study revealed the significant influence of individual perceptions towards COVID-19 vaccines, which were shaped by various social determinants. In the Philippines, socioeconomic disparities are prevalent with a significant portion of the population living below the poverty line [49]. A survey conducted by the Social Weather Stations in March 2023 indicated that 51% of Filipino families, equivalent to around 14 million families, considered themselves poor [50]. Nearly 18.1% of the population (approximately 19.99 million individuals) were living below the poverty line [51]. These socioeconomically disadvantaged populations may encounter barriers to receiving vaccines due to limited access to healthcare, lack of transportation, and inadequate information. One systematic review confirmed our findings that socioeconomic status impacted vaccine equity, with the poorest 27% less likely to be vaccinated compared to the richest group. Furthermore, children whose mothers had no formal education had a 27% lower likelihood of being fully vaccinated compared to those whose mothers had completed primary education or higher [25]. In addition to the socioeconomic status, exposure to misinformation amplified by social media and social networks within the community (such as family members, relatives, and pastors) significantly impacts an individual’s perception of vaccination and thus vaccine uptake [26, 52].

Our study also found a strong link between individuals’ perceptions of COVID-19 vaccines and their trust in the government. Historically, Filipinos have exhibited high levels of vaccine confidence, with rates reaching up to 93% of the population in 2015 [53]. However, a survey conducted by the Social Weather Stations in 2021 indicated that only 45% of the population was willing to take a COVID-19 vaccine and 21% refused to take it [54]. One significant turning point leading to the dramatic decline in public vaccine confidence was the dengue vaccine controversy in 2017, which has resulted in deaths and serious illnesses among children [53, 55]. This incident, constantly brought up by our participants, has led to a series of ripple effects, eroding trust in the government, driving people to seek health information from alternative sources, and contributing to vaccine hesitancy. Therefore, rebuilding public trust in the government and vaccine campaigns is of utmost importance to ensure effective vaccine delivery during public health crises. This requires transparent communication, clear dissemination of accurate information, and proactive efforts to address concerns and doubts. Government should also engage in open dialogue with the public, provide comprehensive explanations of vaccine safety, efficacy, and benefits, and collaborate with community-based partners to conduct health education initiatives to encourage widespread vaccine uptake [56–58].

Our findings suggest that the Health Equity Implementation Framework is valuable for understanding vaccine equity determinants, as it helps to identify and analyze a wide range of factors influencing vaccine access and uptake. However, since it was primarily developed in a clinical context, it has limitations when applied to global health. Specifically, constructs related to providers and organizational contexts may not be relevant on a global scale. This indicates a need for adaptation of this framework to better suit global health applications. To date, there have been a number of frameworks developed to guide the equitable COVID-19 vaccine allocation [59–61]. One notable framework is the COVID-NEEDS framework to guide the assessment of factors that need to be considered to inform the equitable global allocation of vaccines [62]. This framework encompasses ten factors from the social, economic, and health system domains, such as clinical vulnerability, outbreak response systems, virological features, incidence, and spread. Our study aligns with the factors in this framework, particularly the importance of understanding COVID-19 dynamics and health system resilience at the contextual domain, as well as the influence of socioeconomic status on recipients’ vaccine confidence and hesitancy. However this COVID-NEEDS framework was developed primarily for global vaccine allocation and may not fully capture the national and local specific contexts. In contrast, our study identified Philippines-specific contexts, such as unique geographical disparities and religious beliefs among populations, which impacted equitable vaccine distribution. Another noticeable framework is the one developed by the National Academies of Sciences, Engineering, and Medicine in the United States to guide their national-level equitable vaccine distribution [63]. This framework illustrates the overarching goal of vaccine distribution as reducing severe morbidity and mortality and negative societal impact, which informs the allocation criteria and allocation phases. The framework is underpinned by two foundational principles: ethical principle (i.e., maximum benefit, equal concern, and mitigation of health inequities) and procedural principle (i.e., fairness, transparency, and evidence-based). While this framework was developed in the United States context, its overarching goals, allocation criteria, and phases, as well as the foundational principles could also apply to the Philippines [64]. Our findings complement this guiding framework with action-oriented purposes by identifying specific implementation barriers and strategies for equitable vaccine delivery.

Based on the findings of this study, we offer three major recommendations to enhance equitable vaccine delivery that can be applied to other LMICs to prepare for future public health emergencies. First, the findings underscore the need for sustained investments in strengthening the health system to ensure its resilience and sustainability in combating future health emergencies. This entails improving infrastructures, enhancing capacities, and practicing effective governance and coordination mechanisms to enable efficient vaccine distribution. Second, understanding and addressing public vaccine hesitancy is imperative for successful vaccine distribution campaigns. Engaging with communities, providing channels for accessing reliable information, and addressing concerns and doubts are critical steps to build trust and increase vaccine acceptance. Tailored communication strategies that consider cultural, linguistic, and socioeconomic factors can help address vaccine hesitancy and foster vaccine confidence among different population groups. Third, from a policy formulation and implementation perspective, governments need to establish formal guidelines for emergency vaccine deployment and distribution in the future. This includes developing protocols for the rapid development, approval, and distribution of vaccines in response to emerging pandemics. Emphasis should be placed on ensuring transparent communication strategies and implementing robust supply chain management that prioritizes equitable access to vaccines for all populations, especially marginalized and vulnerable groups.

For future research, the lessons learned from COVID-19 vaccination practices can significantly inform our future vaccination strategies. We can develop and evaluate the effectiveness and cost-effectiveness of strategies aimed at improving vaccination rates among marginalized and vulnerable populations. Specific implementation approaches, such as community engagement and the use of vaccine champions, should be tested to combat misinformation, enhance vaccine trust, and increase vaccination rates.

### Limitations

There are several limitations in this qualitative study. First, due to the resource availability limitations during the pandemic, our study participants were primarily from the province of Negros Occidental the Philippines, which may limit the transferability of our findings to other regional contexts in the Philippines, and more broadly LMICs. Second, although we identified various factors at different domains that influenced vaccine delivery, we did not extensively explore the complex relationships, such as moderator or mediator roles, among these factors. Understanding the intricate interplay and dynamics between these factors would provide a more comprehensive and global picture of the influencing factors in vaccine delivery. Third, it is important to acknowledge that our study was conducted during a later stage of the COVID-19 vaccine rollout in the Philippines. As a result, some of the initial challenges during the early stages of vaccine distribution may have been addressed or mitigated. The perspectives and experiences shared by participants may have been influenced by the progress made in addressing those challenges.

## Conclusion

By applying the Health Equity Implementation Framework, we examined factors that influenced equitable vaccine delivery in the Philippines at the context, innovation, and recipient domains. While some factors were specific to the Philippines, such as geographical disparities and religious beliefs among ethnolinguistic groups and individuals, other factors, such as vulnerable health systems and vaccine hesitancy due to socioeconomic status and exposure to misinformation (particularly on social media), were common challenges faced by other LMICs. The findings highlighted the need for LMICs to have sustained investments in strengthening the health system resilience and sustainability in preparing for future health emergencies, and use multilevel strategies to address public vaccine hesitancy to improve vaccine uptake and coverage. Moreover, each LMIC must be attentive to its unique local context to develop tailored implementation strategies to promote equitable vaccine distribution.

## Electronic supplementary material

Below is the link to the electronic supplementary material.

Supplementary Material 1

## Data Availability

The dataset (which includes individual transcripts) is not publicly available due to confidentiality policies. Readers can reach XW for the dataset upon reasonable request.
