# Supplementary material for: Vaccine equity implementation: exploring factors influencing COVID-19 vaccine delivery in the Philippines from an equity lens
Source: BMC Public Health. 2024 Nov 5;24:3058. doi: 10.1186/s12889-024-20578-7 (PMC11539703; doi:10.1186/s12889-024-20578-7)
Supplement: Supplementary file 1 — Supplementary Material 1 [file 12889_2024_20578_MOESM1_ESM.docx]

**Policy Makers- responses will be focussed on policy-level perspective**

| Demographic Information | | | |
| --- | --- | --- | --- |
| DATE:  (mm/dd/yy) |  | DIVISION/CATCHMENT AREA/FACILITY: |  |
| TYPE OF INTERVIEW: (tick) | - POLICY - HEALTH FACILITY - COMMUNITY | UNIQUE ID: |  |
| FACILITATOR: |  | NOTE TAKER: |  |
| START TIME: |  | FINISH TIME: |  |

Introductory questions

1. Can you please tell us about your name, age, highest education, occupation and position?
2. Can you also tell us about your role in COVID-19 vaccine roll out? What are your main responsibilities specifically?

Main questions

1. The Philippines government published the National deployment and vaccination plan for COVID-19 vaccines in January 2021. Were you involved in developing this guideline? 🡪 If yes, could you please tell us more about how was this guideline developed and which department/agencies were involved in developing the national guideline? 🡪 if no, do you know anything about how this guideline was developed (eg. which agencies/departments were involved)?
2. Currently about 70 million people completed 2 doses (around 70% of total population), and only 15 million received a booster dose. Are there any targets set by the government for 2 doses and booster dose?
3. Please describe the process of how the public receives COVID-19 vaccination (vaccination drives)?
4. How does the government secure vaccine supplies?
5. How are vaccines prioritized for distribution when supplies are limited?
6. What factors do you think that helped COVID-19 vaccine roll out in the Philippines?
7. What are the challenges to improving vaccination rate in the Philippines?
8. What do you see as the main barriers to vaccine acceptance and uptake by the public?
9. What are the strategies used to improve vaccine acceptance and uptake in the public? Can you give examples?
10. Apart from essential workers and vulnerable groups (such as elderly and indigenous people) that are prioritized for vaccination, do you think there are other groups that are also vulnerable during the pandemic and may need special attention and help to get vaccinated?
11. What do you think are the main barriers to vaccine acceptance and uptake among **vulnerable groups**?
12. Do you believe the current policy supports equitable vaccination outcomes? Why or why not?
13. What strategies do you think could be used to improve vaccine acceptance and uptake among vulnerable populations?

**Administrators and Health Workers**

| Demographic Information | | | |
| --- | --- | --- | --- |
| DATE:  (mm/dd/yy) |  | CATCHMENT AREA/FACILITY: |  |
| TYPE OF INTERVIEW: (tick) | - POLICY - HEALTH FACILITY - COMMUNITY | UNIQUE ID: |  |
| FACILITATOR: |  | NOTE TAKER: |  |
| START TIME: |  | FINISH TIME: |  |

Introductory questions

1. Can you tell me about your age, highest education, occupation and position, responsible for which facilities?
2. Can you tell me about your main responsibilities in COVID-19 vaccine roll out specifically?

Main questions

1. Can you please describe the process of how the public receives COVID-19 vaccination?
2. Have you heard of any national vaccination policy, guideline or plan? 🡪 If yes, continue with questions. 🡪 If not, skip to Q. 5.
3. Can you explain the process of how vaccine supplies are distributed in your locality?
4. How are vaccines prioritized for distribution when supplies are limited?
5. How do you report vaccination progress of your vaccination site?
6. How are questions, complaints or feedback of the public managed?
7. What factors do you think helped COVID-19 vaccination roll out in the Philippines?
8. What are the challenges to improve vaccination rate in the Philippines?
9. What do you see as the main barriers to vaccine acceptance and uptake by the general public?
10. Where do people usually get information about COVID-19 vaccines?
11. What are the strategies used to improve vaccine acceptance and uptake in the public? Can you give examples?
12. Apart from essential workers and vulnerable groups (such as elderly and indigenous people) that are prioritized for vaccination, do you think there are other groups that are also vulnerable during the pandemic and may need special attention and help to get vaccinated?
13. What do you think are the main barriers to vaccine acceptance and uptake among **vulnerable groups**?
14. Under the current vaccination policies and engagement strategies, do you believe vulnerable populations have the same opportunities to be vaccinated as the general public? Why or why not? (Vulnerable population: individuals who have higher COVID infection risks and those who have disadvantages in accessing health services because of their race, income, gender, etc.)
15. What do you think could be done to improve vaccine acceptance and uptake among vulnerable populations?

**Interview guide – community member**

| Demographic Information | | | |
| --- | --- | --- | --- |
| DATE:  (mm/dd/yy) |  | CATCHMENT AREA: |  |
| TYPE OF INTERVIEW: (tick) | - POLICY - HEALTH FACILITY - COMMUNITY | UNIQUE ID: |  |
| FACILITATOR: |  | NOTE TAKER: |  |
| START TIME: |  | FINISH TIME: |  |

1. Can you please tell us about your age, occupation, and highest education?
2. How many vaccines did you receive so far?
3. What were your motivations to get vaccinated?
4. Did you feel any hesitation or worry initially to get the vaccine?
5. Can you please tell us the process of how you received COVID-19 vaccinations? For example, you can start from how you registered?
6. What elements about the process do you think were convenient (that you liked)?
7. What element do you think were inconvenient or unpleasant (that you didn’t like)? (e.g., long queue, vaccination sites too far, shortage of vaccine supplies)
8. Did you face any resistance from friends or family members who did not support you getting the vaccine?
9. In your community, what are the general perceptions towards COVID-19 vaccines?
10. In your community, are most people willing to get vaccinated and have already received 2 doses?
11. Do you know anyone in your family or community who have not been vaccinated?
12. Do you know anyone in your family or community who have not receive a third/booster dose?
13. Where did you learn about COVID-19 vaccines and information about where and how you can get vaccinated?
14. Did you receive reminders to go for your vaccine?
15. Are there any other activities from the government or organizations that encourage or motivate people to get vaccinated?
16. Is there anything that you would like to understand better about the vaccine or process to receive it?
